# Myocardial Infarction With Ventricular Wall Aneurysm: A Case Report

**DOI:** 10.7759/cureus.29017

**Published:** 2022-09-11

**Authors:** Raviprakash Meshram, Vikas Vaibhav, Shruti Agrawal, Gitanjali Khorwal, Kshitiza Sharma

**Affiliations:** 1 Forensic Medicine, All India Institute of Medical Sciences, Rishikesh, Rishikesh, IND; 2 Forensic Medicine and Toxicology, All India Institute of Medical Sciences, Rishikesh, Rishikesh, IND; 3 Pathology, All India Institute of Medical Sciences, Rishikesh, Rishikesh, IND; 4 Anatomy, All India Institute of Medical Sciences, Rishikesh, Rishikesh, IND; 5 Mental Health Nursing, All India Institute of Medical Sciences, Rishikesh, Rishikesh, IND

**Keywords:** pseudoaneurysm, myocardial infarction, false aneurysm, true aneurysm, ventricular wall aneurysm

## Abstract

A ventricular aneurysm can be pseudo or true; it is a rare complication of myocardial infarction induced by an intra-myocardial dissecting hematoma due to fragile myocardium. Ventricular wall rupture takes place two to four days after myocardial infarction when coagulative necrosis and inflammatory infiltrate and lysis of myocardial connective tissue results in weakening of infarcted myocardium. Acute cardiac wall ruptures are mostly fatal; an unwittingly located pericardial adhesion can abort a rupture resulting in a false aneurysm. The wall of a false aneurysm consists of the epicardium in contrast to a true aneurysm, which is composed of the myocardium. True aneurysms are complications seen in transmural infarcts. Thinned-out scar tissue paradoxically bulged during systole, and toughened fibrotic wall rupture doesn't usually occur. Deaths in subjects with true ventricular aneurysms occur due to mural thrombus, arrhythmias, and heart failure. We encountered a case of a true aneurysm, as reported below.

## Introduction

A ventricular aneurysm is a dreaded myocardial infarction complication that develops in post-myocardial infarction's remodeling phase. There can be true aneurysms and false aneurysms; the latter constitute only of pericardial layer; it lacks myocardial and epicardial elements [1]. It has been observed that the incidence of true aneurysms of the heart following myocardial infarction is approximately 10% [2]. Aneurysms exhibit either paradoxical systolic expansion, total akinesis, or both [3]. Transmural myocardial infarction results in the weakening of ventricular walls leading to ventricular aneurysms. Other causes of a false aneurysm can be cardiac surgery, chest trauma, congenital heart disease, and tumor invasion [1,4]. Presenting symptoms range from chest pain, palpitations, dyspnea, and syncope to nonspecific symptoms, and up to 10% may be asymptomatic [4]. The presenting symptoms in false aneurysms are nonspecific, and diagnosis is often delayed. We present a case of a male with a true ventricular aneurysm found dead inside his room.

## Case presentation

A 32-year-old male with an alleged history of being found down at his residence on Mar 15, 2021, at around 1:00 AM by co-workers at Timli, Tehri Garhwal, Uttarakhand. He used to work with a construction company as a laborer at the construction site. He was ill on and off and used to take some medicines from the local medical shop, as told by his co-workers. The subject belonged to low socioeconomic status and had no recorded past medical or surgical history.

**Figure 1:**
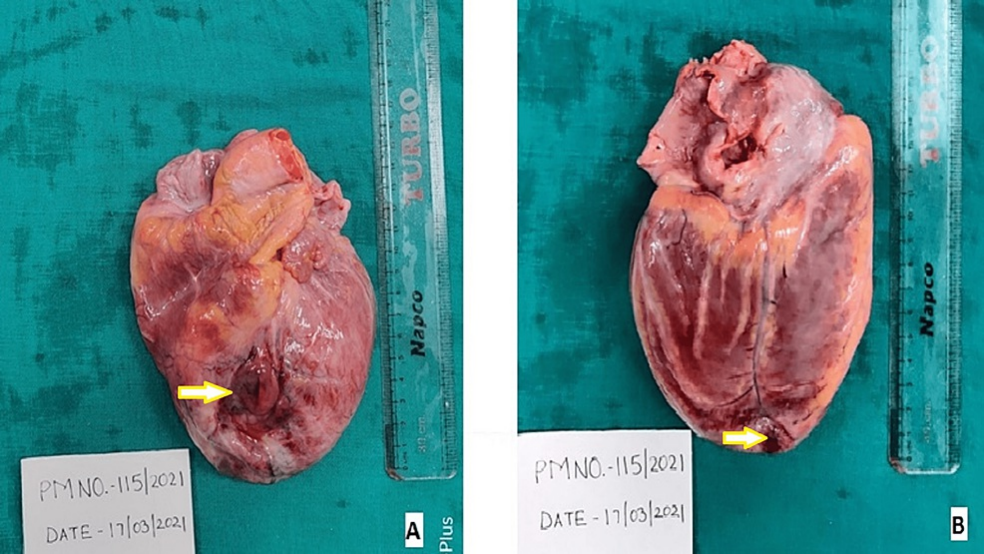
The anterior and posterior surfaces of the heart A: the anterior surface of the heart shows the aneurysm, as shown by the arrow. B: the posterior surface of the heart shows petechial hemorrhages, as shown by the arrow.

On post-mortem examination, the body was a moderately built male with a congested face and eyes with cyanosis over fingernails. On dissection, the lungs and thoracic cavity showed 400 ml of straw-colored fluid on the right side and 390 ml on the left side. The right lung weighed 720 gm and the left - 460 gm; both the lungs were congested, and on the cut section, pinkish frothy fluid was present in both lungs. The pericardium was intact; 210 ml of serous thin pinkish fluid was present on the opening. The heart weighed 460 gm. Grossly enlarged heart with thinning out of the anterior wall over the middle part of the interventricular septal area with a diameter of 3 cm (Figure 1A) was surrounded by ecchymotic patches and petechial hemorrhages over the anterior, anterolateral, and posteroinferior surface (Figure 1B). There were multiple petechial hemorrhages at places over the surface of the heart. Coronaries showed left anterior descending artery with atheromatous changes with calcification causing >98% stenosis, 1.5 cm distal to origin. The right coronary artery showed atheromatous changes with calcification causing 95% stenosis. The left circumflex artery showed athermanous changes with calcification causing 60-70% stenosis.

**Figure 2:**
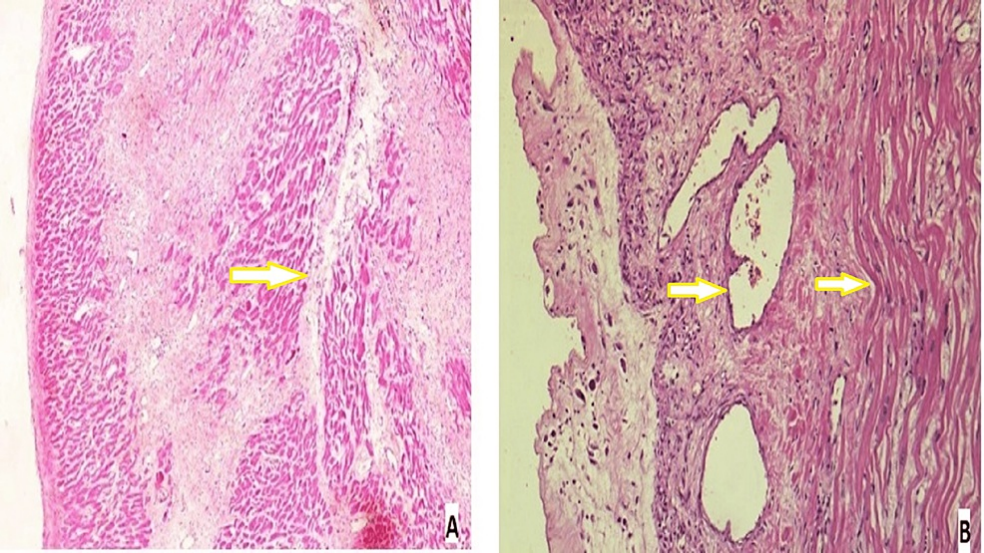
Histological examination of the left ventricular bulge A: left ventricular bulge showing thinned-out myocardium with loss of muscle fibers at places (H&E, 100x), as shown by the arrow. B: higher magnification shows endomyocardium with dilated vessels and haphazard myocardial arrangement (H&E, 100x), as shown by the arrow.

On histological examination, features of myocardial infarction over the left ventricle wall with severe atherosclerosis of the left anterior descending artery were present. The left ventricular bulge showed thinned-out myocardium with a loss of muscle fibers at places, suggesting a true ventricular aneurysm (Figure 2A). Higher magnification shows endomyocardial with dilated vessels, and haphazard myocardial arrangement as the heart is in the remodeling phase (Figure 2B).

**Figure 3:**
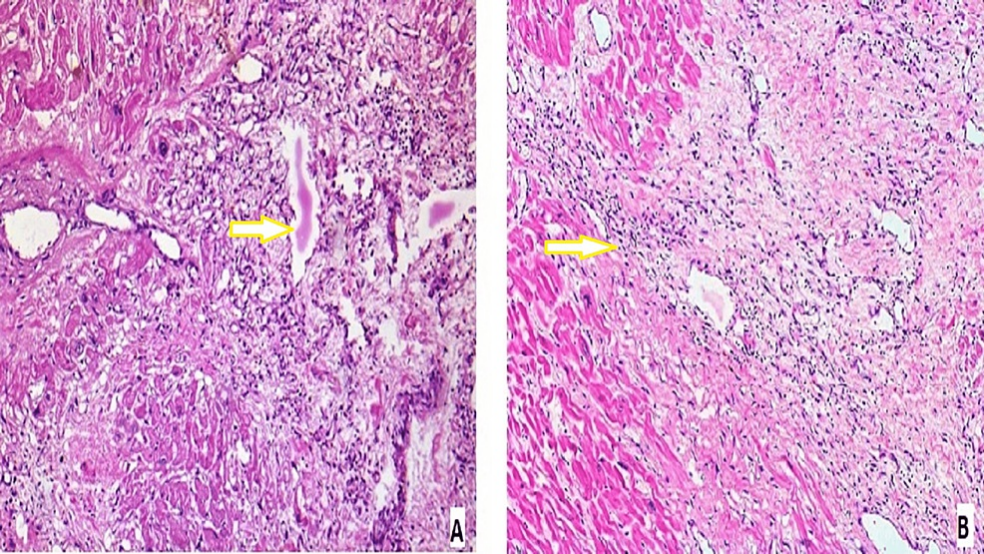
Necrosed myocardiocytes and dense inflammation and edema A: necrosed myocardiocytes with fibrosis and granulation tissue areas (H&E, 100x), as shown by the arrow. B: dense inflammation and edema within a ventricular wall (H&E, 100x), as shown by the arrow.

There were necrosed myocardiocytes with areas of fibrosis and granulation tissue (Figure 3A). Histological examination also revealed dense inflammation and edema within a ventricular wall (Figure 3B).

**Figure 4:**
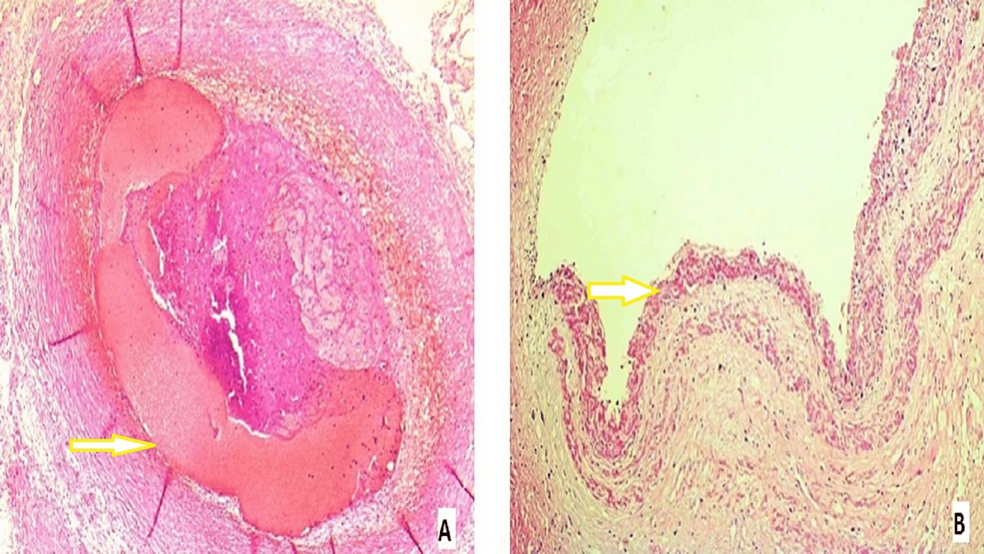
A cross-section of the left anterior descending artery and left coronary artery A: cross-section of the left anterior descending artery with luminal occlusion by thrombosis and atheroma (H&E, 100x), as shown by the arrow. B: partial atherosclerosis of the left coronary artery (H&E, 100x), as shown by the arrow.

A cross-section of the left anterior descending artery and left coronary artery showed luminal occlusion by thrombosis and atheroma (Figure 4).

## Discussion

A restricted hematoma dissecting into a transmural infarct is a reason for a false aneurysm. Through a tiny aperture, it interacts with the left ventricle. False aneurysms have a pericardium and mural thrombus wall without recognizable epicardial or myocardial features. Both true and false heart aneurysms are consequences of myocardial infarction, although they can have radically different etiologies, effects on ventricular function, diagnostic findings, complications, pathological findings, and treatment. A genuine ventricular aneurysm is brought on by the gradual weakening of a section of the ventricular wall after a transmural infarction, and the thinned-out area may gradually expand until it significantly outgrows the infarct's initial surface area. The end result is a non-contractile, maybe expansile segment that is a part of the circumferential wall borders of the left ventricle [1]. Pericardium that is adhered to the underlying epicardium makes up the wall of genuine true aneurysms; fibrous connective tissue of the infarcted cardiac muscle frequently lies beneath this. A false aneurysm is formed by hemorrhagic dissection into the wall of transmural infarction [1].

Although a left aneurysm is frequently suspected clinically, often left ventricular cineangiography is typically used to confirm the diagnosis. A fake aneurysm frequently manifests as a distinct cavity that fills through a tiny orifice during systole and returns back to the ventricle during diastole [1]. The myocardium is at a mechanical disadvantage when the size of an actual aneurysm exceeds 20 to 25% of the ventricle area, which can only be overcome by the heart by dilating the ventricular cavity [1,3]. The false aneurysm does not interfere with muscle function but acts to unload the ventricle during systole, almost like bicuspid valve incompetence, which will reduce flow and, indirectly, end in left ventricular dilatation. False aneurysms differ in their gross and microscopic pathology and operative management. Indications for operative intervention are equivalent to those for a true aneurysm; however, since they need a bent toward rupture, which by true aneurysms, prophylactic repair may be a further indication for operation even within the asymptomatic patient [1].

An aneurysm is a rare complication of myocardial infarction. Transmural myocardial infarction is the most common cause of a false aneurysm [4]. The rupture is sealed by pericardial attachments, forming a hematoma, thrombus, or scar tissue, forming an aneurysm [4-6]. If left undiscovered, the fake aneurysm wall, which is only made up of fibrous tissue or pericardium and lacks the true layers of endocardium and myocardium, has a very high mortality rate [4,7]. These aneurysms require prompt detection and treatment because they are more likely to burst [4,6]. Congenital heart disease, chest injuries, tumor invasion, cardiac surgery, and endocarditis are some other etiologies causing aneurysms [4,5,8,9].

The incidence of a false ventricular aneurysm is 0.1% in patients with myocardial infarction and 0.8% in patients who have undergone mitral valve replacement surgery [4,10,11]. The majority of patients were White (75%), and male (67%), and the mean age at presentation was 60 years, according to one study [4,12]. Untreated left ventricular false aneurysms have a 30-45% likelihood of rupturing [4,13].

Although it can develop up to 12 months after a myocardial infarction, a left ventricular false aneurysm typically develops within three to 14 days of the myocardial infarction [4,9]. Increased macrophage activity in the infarcted tissue as a result of the inflammatory process causes remodeling and tissue thinning of the necrotic myocardium. The sealed wall rupture as a false aneurysm at that time as a result of the inflammatory and prothrombotic state [4,14].

Up to 10% of patients may be asymptomatic, and some may present with several vague symptoms. Chest discomfort, palpitations, dyspnea, and syncope are common symptoms in certain people; other patients may also experience indistinct symptoms such as coughing, fever, disorientation, shoulder or back pain, dysphagia, congestive heart failure, and stroke [4,11]. Surgery is the only definitive treatment for left ventricular false aneurysms. According to various research, asymptomatic patients with an accidental diagnosis of a false aneurysm, which was smaller than 3 cm in size, and patients who are not medically qualified for surgery are managed non-surgically [4,11]. Given their ability to resemble false aneurysms, true left ventricular aneurysms deserve consideration. When compared to false aneurysms, true aneurysms are a very common problem that affects 10% to 35% of patients [4,14]. Preoperatively, it can frequently be challenging to distinguish between aneurysms. A true aneurysm, as compared to a false one, contains all three layers of the myocardial wall and is characterized by contractile dyskinesia or a paradoxical bulge with a left ventricular diastolic contour area, which lowers the ejection rate [4]. True aneurysms usually occur in the anterior or apical region, while false aneurysms more commonly occur in the posterior region. Mostly true, aneurysms are non-contractile and have a thin myocardium [11].

Due to the false aneurysm sac's inherent fragility, it can cause dyskinetic heartbeats that can result in arrhythmias, clot formation, and even heart failure [4,5]. A false aneurysm's common sequelae are the adherent sac's rupture [15].

The likelihood of a left ventricular (LV) pseudoaneurysm increases following an inferior myocardial infarction. Despite typical presentations like heart failure, embolism, and arrhythmias, some aneurysms are unexpectedly stable and go unnoticed for years [16]. Differentiating an LV pseudoaneurysm from a true aneurysm is necessary because rupture can be prevented with early surgical intervention [16]. 

## Conclusions

Among all the late complications of acute myocardial infarction, a left ventricular wall aneurysm is uncommon and possibly like a hidden bomb, so one must be vigilant to the presenting symptoms related to cardiac performances or any sudden change in the hemodynamic stability of the patient. Advances in radiological imaging have increased the capacity to differentiate cardiac pseudoaneurysms from different pathologies. Most pseudoaneurysms, specifically if acute or related to symptoms, require surgical treatment to lessen the chances of rupture; if treated adequately and promptly, it avoids sudden deaths.
